# Validity of the Alberta Infants Motor Scale in Norwegian infants aged 6–9 months through comparison with Canadian and Dutch scores

**DOI:** 10.3389/fped.2024.1511965

**Published:** 2025-01-14

**Authors:** Anniken Göthner, Kirsti Riiser, Kine Melfald Tveten

**Affiliations:** ^1^ Department of Child and Adolescent Health Promotion Services, City District of Vestre, Oslo, Norway; ^2^ Department of Rehabilitation Science and Health Technology, Faculty of Health Sciences, Oslo Metropolitan University, Oslo, Norway; ^3^ Department of Child and Adolescent Health Promotion Services, Norwegian Institute of Public Health, Levanger, Norway; ^4^ Department of Health and Functioning, Faculty of Health and social science, Western Norway University of Applied Sciences, Bergen, Norway; ^5^ Children’s Physiotherapy Center, Bergen, Norway

**Keywords:** infant, motor development, assessment, validity, Alberta Infant Motor Scale

## Abstract

**Introduction:**

The Alberta Infant Motor Scale (AIMS) is widely used to assess infant motor development but has shown limited cross-cultural validity in various populations. The distribution of the original AIMS scores has not been cross-culturally validated for Norwegian infants. This study aimed to evaluate the applicability of the Canadian AIMS norm reference for Norwegian infants aged 6–9 months and compare their percentile rankings with the Canadian and Dutch norms.

**Methods:**

In this cross-sectional study, AIMS scores from a sample of 189 Norwegian infants aged 6–9 months were compared to the Canadian and Dutch norms. Total raw scores from the Canadian norms were compared to those of the Norwegian sample, and the percentiles of the Canadian and Dutch sample were compared to tentative Norwegian percentiles.

**Results:**

Norwegian infants aged 6–9 months consistently scored lower on the AIMS than their Canadian counterparts (*p* < 0.001), with 81% scoring at or below the 50th percentile and 18% falling at or below cut-off indicating possible motor delay. Using the Dutch norms, 20% of the Norwegian sample scored at or below the 50th percentile, while only 1% scored at or below the cut-off. A comparison of the percentile ranks showed that Canadian norms had the highest ranks for all age groups, followed by the Norwegian sample and subsequently the Dutch norms. The observed difference is considered clinically significant.

**Conclusion:**

Neither Canadian nor Dutch AIMS norms are valid for Norwegian infants due to the Canadian norms being too stringent and the Dutch norms being too lenient. A thorough cross-cultural validation for infants 0–18 months to establish Norwegian-specific AIMS norms is recommended.

## Introduction

A thorough understanding of infant motor development is fundamental to the practice of pediatric physiotherapists. Infant motor development encompasses the development of postural control, movement patterns, and coordination, which are essential for the acquisition of motor skills. The complex and dynamic process of infant motor development is affected by several factors, including genetic, physical, neurological, social, and environmental factors (1). As infants grow, their movement repertoires expand and adjust in response to the challenges and learning opportunities presented by changing environments and task-specific contexts (2). These factors contribute to shaping the trajectory and speed of infant motor development (3).

Assessment tools offer comprehensive insights into infant motor development and are frequently used by pediatric physiotherapists alongside clinical observations (4, 5). Reliable, validated tools are recommended for consistent administration and scoring (6, 7). These tools help identify atypical or delayed motor development early, ensuring only infants in need receive interventions, thus preventing unnecessary treatment for typically developing infants (8, 9).

It is important to consider possible differences in pace of motor trajectories when using an assessment tool outside its original context (10–12). Hence, part of cross-cultural validity may entail an adaptation of the scores to the cultural context, and development of standards. This approach helps prevent insufficient or excessive follow-up due to invalid norms (13), thereby striking a balance that promotes accurate monitoring and appropriate intervention for each infant's unique developmental pathway.

The Alberta Infant Motor Scale (AIMS) is a widely used standardized tool for identifying infants with atypical or delayed motor function, also in Norway (5, 9, 13). A systematic review found the AIMS to have limited cross-cultural validity in terms of different trajectories for motor development across cultures, with several studies suggesting that the Canadian norms were excessively strict (13). Alternative AIMS reference values from Brazilian, Dutch, Polish, and Thai infants have been introduced to provide more culturally appropriate standards (14–17). These studies showing that the AIMS has low cross-cultural validity underline the need to examine whether the Canadian norms are valid for Norwegian infants.

Investigating the validity of the AIMS within the Norwegian infant population is important as the AIMS, with Canadian norms, is currently the most used assessment tool by Norwegian pediatric physiotherapists in primary- and specialist healthcare (5). Findings suggest that Norwegian children start walking independently significantly later than the Canadian AIMS norm reference (18). Additionally, Norwegian infants are found to achieve gross motor milestones later than other populations (3) all of which supports the need to investigate validity.

In this article, the cross-cultural validity of the AIMS will be investigated by comparing the scoring distributions. The primary aim of this cross-sectional study was to investigate the cross-cultural validity of the AIMS Canadian norm reference for Norwegian infants aged 6–9 months. The secondary aim was to compare the Norwegian sample's AIMS scores with those of the Dutch norms for infants in the same age range. The choice of Dutch norms for comparison stems from the geographical proximity and cultural similarities between the Netherlands and Norway.

The 6–9-month age interval range was targeted in this study because the most compelling evidence for identifying delayed motor function with the AIMS typically appears after eight months of corrected age (9). In this age range, infants are likely to be assessed with more items than in the younger and older age groups. Moreover, other studies on the cross-cultural validity of the AIMS have included this age range (14–17, 19–25), which allows for comparison. This phase is also crucial for infants as they refine their muscle coordination, heavily influenced by explorative activities that are essential for motor development (26). Additionally, the largest number of infants in the available sample was in the age range 6–9 months.

This investigation serves as a preliminary investigation, and the findings of this study will indicate whether there is a need for a comprehensive cross-cultural validation of the AIMS across all age groups in Norway.

## Materials and methods

### Design, participants and recruitment

The data of Norwegian infants used in this cross-sectional study was extracted from a previous study on infant motor assessment (27). The inclusion criteria were infants aged 3–18 months, corrected for prematurity. The exclusion criteria were infants with severe medical conditions that precluded assessment, and those whose parents did not speak and understand Norwegian or English. Data from all infants aged 6–9 months were extracted for this study.

The sample was recruited from four municipalities in western and southeastern Norway; Porsgrunn, Bamble, Tønsberg, and Bergen, between October 2015 and June2020. Public health nurses assisted in recruiting all eligible parents or legal guardians of infants during regular checkups in well baby clinics. Participants were also recruited through word of mouth from former participants.

Detailed informed consent was provided to establish predictability and ensure that parents were fully aware of the study's aim, their role, and the handling of their data (27). The consent letter assured parents that they and the public health nurse would be notified of any concerns regarding the infant's motor function identified during the assessment. Parents were also informed, verbally and in writing, of their right to withdraw from the project at any time without affecting the follow-up service provided by the child healthcare centers. No participants withdrew from the study.

### Method of data collection

Scoring based on the Canadian AIMS norm reference was conducted in first half of 2023 using video recordings of assessments performed between October 2015 and June 2020 (27, 28). The third author (KMT), a specialist in pediatric physical therapy, conducted all assessments. Each infant was assessed once with minimal physical handling, ensuring they were in an alert, non-crying state. Various settings were chosen for conducting the assessments, including well baby clinics, the infant's home, the Western Norway University of Applied Sciences, and the Children's Physiotherapy Center in Bergen. Demographic characteristics of the sample and general population of Norwegian infants were obtained from the Medical Birth Registry of Norway (27, 29).

### Measures

The main variables of interest in this study were the AIMS scores from the Canadian, Dutch, and Norwegian samples (9, 17). The AIMS assesses infants from term (40 weeks gestation) to 18 months post-term, based on observation of qualitative and functional aspects of spontaneous movement (9). Assessment is conducted using 58 observational items in the prone, supine, sitting, and standing positions. Each item is scored as 1 (observed) or 0 (not observed), with a total possible score of 58 points. Age-specific norms, adjusting for corrected age for preterm birth (before 37 weeks gestation), are used for identifying potential motor delays by applying cut-off percentiles. The cut-off for infants up to 8 months is the 10th percentile, and the 5th percentile is used from 8 months onwards (9). The AIMS is considered a cost and time-effective tool with robust psychometric properties (4, 9).

A previous study on the cross-cultural validation of the AIMS norms established a threshold for clinically significant differences as a variation of two points in the raw score (20). This threshold was also applied in this study.

### Statistical analysis

Background characteristics of the Norwegian sample were analyzed using descriptive statistics. To determine the sample's representativeness of the Norwegian infant population, we compared it with open-access data from the Medical Birth Registry of Norway. The chi-square test was applied to assess differences in the categorical variables, while an independent *t*-test was utilized for the continuous variables. The sample distribution on the Canadian and Dutch AIMS percentiles was analyzed using descriptive statistics to calculate frequencies.

The total raw AIMS scores from the Canadian norms were compared to those of the Norwegian sample using an independent *t*-test for each age group, with a statistically significant level set at *p* < 0.05.

The same comparison with the Dutch norms was not possible because the total raw AIMS scores for the Dutch sample were not available (17). However, the Dutch material did provide a table of scores corresponding to each percentile in the norms, a format also available in the Canadian material (9). To facilitate comparison, we created a similar table for the Norwegian sample. This was done by sorting the infants by month and ranking them according to their AIMS scores, we determined the number of points required for the 5th–90th percentiles in the Norwegian sample.

Statistical analysis was performed using IBM SPSS Statistics (Version 29) and Microsoft Excel. Additionally, a medical statistician was consulted to ensure the robustness and validity of the statistical process employed.

### Ethical considerations

This study utilizes previously collected data, where ethical approval was obtained from the Regional Committee for Medical and Health Research Ethics (2016/566 REK vest) and by the Norwegian Social Science Data Service (project no. 45014/3/MSS) (27). Informed consent was obtained from all parents or legal guardians involved.

## Results

### Sample characteristics

Detailed characteristics of the sample, including information about infants and their mothers, are presented in Table 1. The sample comprises 189 infants, distributed across each age interval with a range of 44–51 infants per group. In the group of 9-month-old infants, there was a larger proportion of males, preterm infants, young mothers and variation of birth weight. None of the preterm infants were extremely preterm or had a very low birthweight (Table 1). They were thus considered low-risk infants.

**Table 1:** Background characteristics of the Norwegian sample, infants aged 6–9 months^a^.

	6 month, *n* = 47	7 month, *n* = 51	8 month, *n* = 47	9 month, *n* = 44	Total, *n* = 189
	*n* (%)	*n* (%)	*n* (%)	*n* (%)	*n* (%)
Sex, Female	23 (48.9)	28 (54.9)	21 (44.7)	18 (40.9)	90 (47.6)
GA, weeks, *Mean (SD)*	40 (1.47)	40 (1.41)	39 (0)	40 (0.7)	40 (0.7)
Preterm (<37 weeks GA)	2 (4.4)	0 (0.0)	1 (2.2)	4 (9.3)	7 (3.8)
Birth weight, grams, *Mean (SD)*	3,666 (447.7)	3,701 (436.2)	3,550 (437.3)	3,658 (712.4)	3,644 (515.7)
*n missing=*	*2*	*1*	*1*	*1*	*5*
APGAR 5 min, *Median [min, max]*	10 [8–10]	10 [2–10]	10 [6–10]	10 [6–10]	10 [2–10]
*n missing=*	*2*	*1*	*1*	*1*	*5*
Maternal age, >25 years	0 (0.0)	4 (7.8)	1 (2.1)	4 (9.1)	9 (4.8)
Parity, 0 first-time giving birth	29 (61.7)	29 (56.9)	17 (36.2)	21 (47.7)	96 (50.8)
*n missing=*				*5*	*5*
Married registered partner	42 (93.3)	48 (96.0)	45 (97.8)	42 (97.7)	177 (96.2)
*n missing=*	*2*	*1*	*1*	*1*	*5*

APGAR 5, score of appearance, pulse, grimace, activity, and respiration 5 min after birth (min score = 0, max score = 10); Parity > 0, first time giving birth; Preterm, born before week 37 gestation age (GA); SD, standard deviation; ^#^=, missing values: *n* = 5.

^a^ Data in Table 1 are based on a previous study on infant motor development (27).

No significant differences were found regarding infant sex, preterm birth status, or maternal marital status when comparing the sample characteristics to those of the Norwegian infant population. Significant differences in maternal age and parity were observed; our sample included a smaller percentage of mothers under the age of 25 and a higher percentage of first-time mothers. Additionally, the sample showed a significantly higher average birth weight than the general population. Overall, the sample was considered a low-risk group with demographics representative of the Norwegian infant population.

### Comparison of percentiles in the Canadian, Dutch, and Norwegian sample

**Figure 1:**
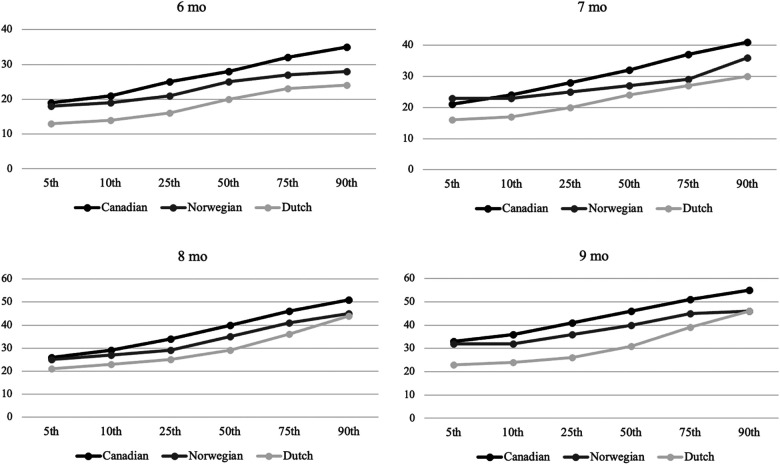
Percentile ranks for Canadian, Norwegian, and Dutch samples aged 6–9 months. *Y*-axis: AIMS total score, *X*-axis percentiles.

Figure 1 presents a tentative Norwegian percentile rank based on the scores of the sample. The Canadian norms consistently show the highest percentile ranks, followed by the Norwegian sample, and then the Dutch norms. The observed differences are considered clinically significant between Canadian and Norwegian norms, and Dutch and Norwegian norms. The Canadian norms are at least two points higher than the Norwegian ranks, with exceptions at the 5th percentile for ages 6, 8, and 9 months, and the 10th percentile for 7-month-old infants. Conversely, the Dutch norms are generally more than two points lower than the Norwegian ranks, except at the 90th percentile for infants aged 8 and 9 months.

### Distribution of Canadian and Dutch AIMS percentiles in the Norwegian sample

**Figure 2:**
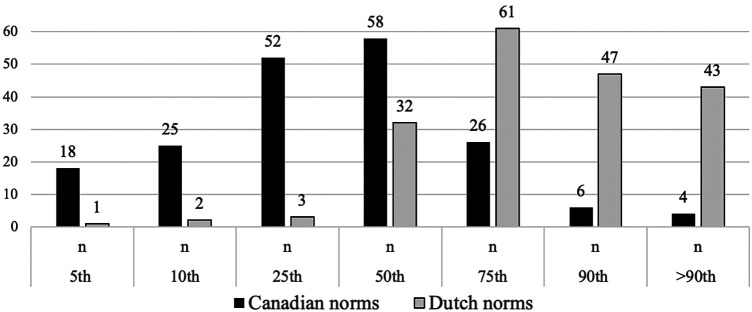
Number of infants aged 6–9 months from the Norwegian sample falling within the Canadian and Dutch percentiles of the Alberta AIMS. *Y*-axis: Number of children, *X*-axis: percentiles.

Using Canadian norms, the median percentile rank for the Norwegian sample is the 50th, and the mean percentile value is 29.8. The Norwegian sample displays a left-skewed distribution with a higher prevalence of lower AIMS scores, as seen in Figure 2. Overall, 81% of the Norwegian sample scored at or below the 50th percentile, with 18% falling at or below the cut-off, indicating a possible motor delay. A substantial proportion of infants across all age groups scored at or below the 50th percentile: 94% at 6 months, 84% at 7 months, 70% at 8 months, and 77% at 9 months. Regarding cut-off scores, 30% at 6 months and 18% at 7 months scored below the 10th percentile. Additionally, 9% at 8 months and 16% at 9 months scored at or below the 5th percentile threshold.

Using Dutch norms, the median percentile rank is the 75th. This indicates a right-skewed distribution with a higher prevalence of higher AIMS scores (Figure 2). Overall, 20% of the Norwegian sample scored at or below the 50th percentile, with only 1% falling at or below the cut-off. Within each age group, a smaller proportion scored at or below the 50th percentile: 23% at 6 months, 22% at 7 months, 28% at 8 months, and 5% at 9 months. Regarding cut-off scores, none of the infants at 6 or 7 months scored below the 10th percentile. At 8 months, 2% fell below the 5th percentile, and at 9 months, no infants scored below this cut-off.

### Comparison of the mean total raw score in the Canadian and Norwegian sample

**Figure 3:**
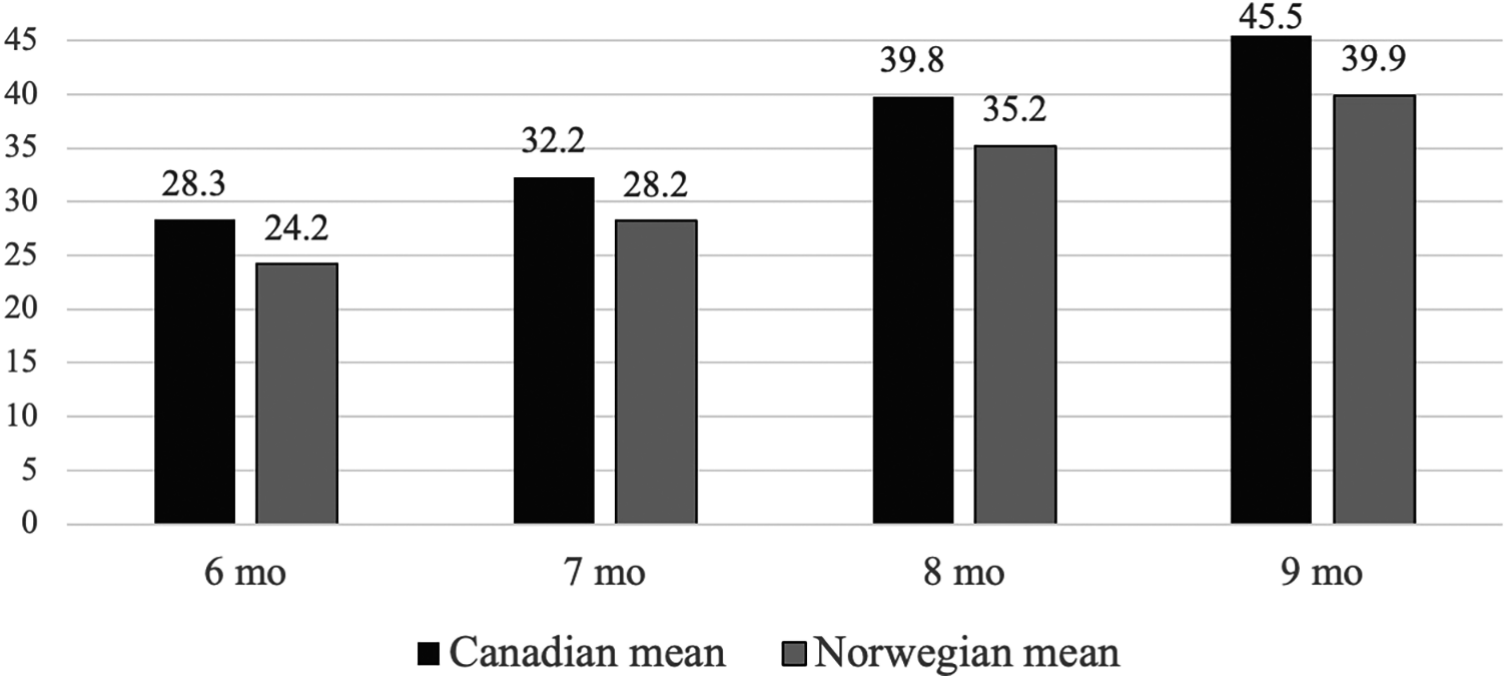
The mean total AIMS score of Norwegian infants and Canadian normative sample aged 6–9 months categorized in monthly intervals.

Figure 3 displays the comparison of the mean total raw AIMS scores between the Canadian normative sample and the Norwegian sample. There is a statistically significant difference across all age intervals with *p*-values below 0.001. The variation in mean total raw scores ranges from 4.1 to 5.6 points.

## Discussion

The primary finding suggests that the Canadian AIMS norm reference is not valid for the sample of Norwegian infants aged 6–9 months. The sample had significantly lower mean total scores than their Canadian peers across all corresponding age groups. This is clinically relevant since the observed differences, ranging from 4.1 to 5.6 points, exceed the two-point threshold for clinically significant differences (20). Additionally, the Canadian AIMS percentile rank was also higher than the Norwegian sample's, further indicating a clinically significant difference.

The secondary finding indicates that the Dutch percentile ranks were lower than those of the Norwegian sample, a difference considered clinically significant due to a general trend of exceeding the two-point threshold.

The sample in the current study mirrors the demographic characteristics of the general population of Norwegian infants, which enhances the generalizability of the results (11). Our findings also align with research suggesting that Norwegian infants' motor skill development pace differs from that of peers in other cultures (3, 18), further lending credibility to our observations.

Our findings support previous research indicating that the AIMS norms are overly strict and have limited cross-cultural validity (14–17, 19–22, 24, 30). A comprehensive cross-cultural validation of the AIMS is warranted to address the observed discrepancies among Norwegian infants.

The findings of this study align with those of a systematic review which indicates that several standardized assessment tools developed in North America may not have universal validity, particularly for assessing motor development in children aged 0–2 years (13). Motor development is known to be influenced by a variety of factors, including biological aspects such as genetics, prenatal health, prematurity, birth complications, physical health, and nutrition, as well as environmental influences like socioeconomic status, environmental stimulation, and psychosocial factors (3, 13, 17).

Cultural caregiving practices are a prominent environmental influence that contribute to differences in motor development (3, 31). Comparing cultural caregiving practices across Canada, the Netherlands, and Norway is challenging due to the subjective nature of cultural norms and their impact on motor development. However, research suggests that cultural norms in North America often promote early sitting and active training (13, 32). In contrast, Norwegian and Dutch cultural norms tend to support a more natural progression, allowing infants to develop at their own pace (3, 17, 18, 31).

Cultural caregiving practices provide diverse experiences, such as positioning and handling routines that encourage movements against gravity (26). These opportunities for motor exploration afforded by the environment and trial-and-error experiences contribute to the acquirement of motor skills (2, 10). North American caregiving practices may potentially lead to an overall faster trajectory of motor development as infants are exposed to positions that require postural control.

On the other hand, the more lenient approach by Norwegian and Dutch caregivers may result in the later attainment of postural control and overall motor development. For example, infants with limited prone position experience often exhibit temporary motor delays, as this position is crucial for developing the upper body strength and motor control needed for movements against gravity (33, 34). These skills are fundamental elements of later motor skills, thereby underlining the influence of early experiences on future motor outcomes (2).

### Strengths and limitations of the study

The sample size in our study was deemed adequate for a preliminary investigation into cross-cultural differences of the AIMS. It is estimated that a minimum of 20 infants per age group can provide 80% power for assessing the cross-cultural validity of the AIMS (35). Additionally, our sample size aligns with those of other studies that have studied the cross-cultural validity of the AIMS (20, 21, 36–39). The sample size was deemed too small to establish reliable percentile ranks (9, 40). Therefore, it is imperative to note that the Norwegian percentile values presented here are provisional and should be interpreted with caution when compared to Canadian and Dutch percentiles. The constrained sample size could potentially introduce bias into the comparison.

The sample was considered a low-risk group with demographics representative of the Norwegian infant population; however, additional examination is necessary. The study's scope was limited to two geographical areas and a few demographic variables. Hence, it is important to acknowledge that these may not encompass all the potential discrepancies between the sample and the broader population. Notably, influential factors such as socioeconomic background and health literacy were not accounted for in the analysis. The potential of self-selection bias ought to be considered, as participants in similar research in Norway tend to have higher socioeconomic background and enhanced health literacy (41–43).

Statistical considerations include the use of an independent samples *t*-test to compare AIMS mean total scores, despite the non-normal distribution of the Norwegian sample. The absence of necessary data for non-parametric tests within the AIMS material (9), necessitated the use of a parametric test for group comparison, which may be considered a study limitation. However, a Mann–Whitney *U* test was also conducted to compare the groups under the assumption that the Canadian values followed a perfectly normal distribution. This assumption introduces yet another potential limitation. Despite these methodological challenges, both tests indicated significant differences between the Canadian and Norwegian samples. After consulting with a medical statistician, we selected the independent samples *t*-test for its alignment with the methods used in other studies that have undertaken cross-cultural validation of the AIMS (14–16, 19, 20, 22, 35, 37, 44, 45). We consider this consistency in methodology as a strength of our research.

### Clinical implications

The stringent Canadian norms, currently used in Norway (5), may lead to the misclassification of normal motor development variations as delays. This could result in unnecessary referrals to pediatric physiotherapy and early intervention services, wasting resources and causing undue stress for families of healthy infants. Conversely, the more lenient Dutch norms might fail to identify infants with genuine delayed motor development among Norwegian infants. This could delay crucial early interventions, necessary service referrals, and the provision of adequate support to families truly in need.

These findings highlight the critical need for validated, culturally appropriate reference values for Norwegian infants to accurately evaluate motor development and prevent misclassification risks. Norwegian reference values should be generated from a large sample that accurately represents the proportion of preterm infants, socioeconomic background, and cultural diversity of the population (46). These considerations are key as these factors are known to influence infant motor development (4, 8, 13, 47–49).

In addition to creating a normative reference for the Norwegian population, it is important to recognize that language and cultural context significantly influence the validity of assessment tools (12). Cultural adaptations must extend beyond mere direct translation and should encompass a thorough translation process of the test manual conducted by a group of experts (50). Such a method ensure that cross-cultural adaptations are tailored to the specific context and are systematically validated, thereby contributing to more accurate assessments that can be reliably used in clinical practice (40).

In the absence of a Norwegian-adapted version of the AIMS, pediatric physiotherapists should cautiously interpret AIMS results, acknowledging that Norwegian infants may exhibit slower motor development compared to Canadian peers. It is important to recognize the limitations of standardized assessments, which might not capture an infant's complete motor skills, potentially leading to discrepancies in observed behaviors (4, 8). The AIMS should be considered one component of a comprehensive evaluation that includes clinical assessments, clinical reasoning, and critical evaluations (5).

## Conclusion

The Canadian and Dutch AIMS norm references are indicated to have limited applicability for Norwegian infants aged 6–9 months in this study, with Canadian norms being too strict and Dutch norms too lenient. A thorough cross-cultural validation to establish Norwegian-specific AIMS norms is recommended.

## Data Availability

The datasets presented in this article are not readily available because Ethical approval presupposes that the data will only be used for the project and the research questions for which approval has been sought. Requests to access the datasets should be directed to Datasets are not available on request due to ethical restrictions.
