# Supplementary material for: Validity of the Alberta Infants Motor Scale in Norwegian infants aged 6–9 months through comparison with Canadian and Dutch scores
Source: Front Pediatr. 2025 Jan 14;12:1511965. doi: 10.3389/fped.2024.1511965 (PMC11772338; doi:10.3389/fped.2024.1511965)
Supplement: Supplementary file 1 [file Datasheet1.docx]

Supplementary:

**Table 2.** AIMS mean raw scores for Canadian and Norwegian samples aged 6 to 9 months^[[1]](#footnote-1)^

|  | Canadian sample | | Norwegian sample | | Comparison of Canadian and Norwegian sample | | | |
| --- | --- | --- | --- | --- | --- | --- | --- | --- |
| Age, mo | n | Mean_C (SD) | n | Mean_N (SD) | Mean_C - Mean_N | 95% CI | P-value | SE of diff |
| 6 | 225 | 28.3 (5.5) | 47 | 24.2 (4.4) | 4.1 | 2.4, 5.8 | <0.001* | 0.9 |
| 7 | 222 | 32.3 (6.9) | 51 | 28.2 (5.8) | 4.1 | 2.0, 6.1 | <0.001* | 1.0 |
| 8 | 220 | 39.8 (8.7) | 47 | 35.2 (7.7) | 4.6 | 1.9, 7.7 | <0.001* | 1.4 |
| 9 | 189 | 45.5 (7.5) | 44 | 39.9 (5.8) | 5.6 | 3.2, 7.9 | <0.001* | 1.2 |

Mean_C=mean raw score of the Canadian normative sample, Mean_N=mean raw score of the Norwegian sample. Values: presented with mean and standard deviation (SD), 95% confidence intervals (95% CI), p-value <0.05 statistically significant*, and standard error of difference (SE of diff). Data in Table 2 are based on previous studies from Canadian (9) and Norwegian (27) samples.

**Table 3.** The AIMS percentile ranks for the Canadian, Norwegian, and Dutch samples aged 6 to 9 months^2^

| Age, mo | Sample | 5th | 10th | 25th | 50th | 75th | 90th |
| --- | --- | --- | --- | --- | --- | --- | --- |
| 6 | Canadian | 19 | 21 | 25 | 28 | 32 | 35 |
|  | Norwegian | 18 | 19 | 21 | 25 | 27 | 28 |
|  | Dutch | 13 | 14 | 16 | 20 | 23 | 24 |
| 7 | Canadian | 21 | 24 | 28 | 32 | 37 | 41 |
|  | Norwegian | 23 | 23 | 25 | 27 | 29 | 36 |
|  | Dutch | 16 | 17 | 20 | 24 | 27 | 30 |
| 8 | Canadian | 26 | 29 | 34 | 40 | 46 | 51 |
|  | Norwegian | 25 | 27 | 29 | 35 | 41 | 45 |
|  | Dutch | 21 | 23 | 25 | 29 | 36 | 44 |
| 9 | Canadian | 33 | 36 | 41 | 46 | 51 | 55 |
|  | Norwegian | 32 | 32 | 36 | 40 | 45 | 46 |
|  | Dutch | 23 | 24 | 26 | 31 | 39 | 46 |

**Table 4.** Distribution of Norwegian infants aged 6 to 9 months on the Canadian and Dutch AIMS percentiles^[[2]](#footnote-2)^

| ***Canadian*** | | 5^th^ | | 10^th^ | | 25^th^ | | 50^th^ | | 75^th^ | 90^th^ | | >90^th^ | |  |
| --- | --- | --- | --- | --- | --- | --- | --- | --- | --- | --- | --- | --- | --- | --- | --- |
| ***norms*** | | n (%) | | n (%) | | n (%) | | n (%) | | n (%) | n (%) | | n (%) | |  |
| 6 mo | | 6 (12.8) | | 8 (17.0) | | 9 (19.1) | | 20 (42.6) | | 3 (6.4) | 0 (0.0) | | 1 (2.1) | |  |
| 7 mo | | 1 (2.0) | | 8 (15.7) | | 19 (37.3) | | 15 (29.4) | | 3 (5.9) | 2 (3.9) | | 3 (5.9) | |  |
| 8 mo | | 4 (8.5) | | 4 (8.5) | | 13 (27.6) | | 12 (25.5) | | 10 (21.3) | 4 (8.5) | | 0 (0.0) | |  |
| 9 mo | | 7 (15.9) | | 5 (11.4) | | 11 (25.0) | | 11 (25.0) | | 10 (22.7) | 0 (0.0) | | 0 (0.0) | |  |
| ***Dutch*** | 5^th^ | | 10^th^ | | 25^th^ | | 50^th^ | | 75^th^ | | | 90^th^ | | >90^th^ | |
| ***norms*** | n (%) | | n (%) | | n (%) | | n (%) | | n (%) | | | n (%) | | n (%) | |
| 6 mo | 0 (0.0) | | 0 (0.0) | | 0 (0.0) | | 11 (23.4) | | 9 (19.2) | | | 3 (6.4) | | 24 (51.1) | |
| 7 mo | 0 (0.0) | | 0 (0.0) | | 1 (2.0) | | 11 (21.6) | | 16 (31.4) | | | 13 (25.5) | | 10 (19.6) | |
| 8 mo | 1 (2.3) | | 1 (2.3) | | 2 (4.3) | | 9 (19.2) | | 17 (36.2) | | | 10 (21.3) | | 7 (14.9) | |
| 9 mo | 0 (0.0) | | 1 (2.3) | | 0 (0.0) | | 1 (2.3) | | 19 (43.2) | | | 21 (47.7) | | 2 (4.5) | |

1. Data based on previous studies from Canadian (9), Norwegian (27) and Dutch (17) samples [↑](#footnote-ref-1)
2. Data based on previous studies from Canadian (9), Norwegian (27) and Dutch (17) samples [↑](#footnote-ref-2)
